# Cooperation and Competition with Hyperscanning Methods: Review and Future Application to Emotion Domain

**DOI:** 10.3389/fncom.2017.00086

**Published:** 2017-09-29

**Authors:** Michela Balconi, Maria E. Vanutelli

**Affiliations:** ^1^Research Unit in Affective and Social Neuroscience, Catholic University of Milan, Milan, Italy; ^2^Department of Psychology, Catholic University of Milan, Milan, Italy; ^3^Department of Philosophy, Università degli Studi di Milano, Milan, Italy

**Keywords:** EEG, emotions, hyperscanning, cooperation, competition, social interaction, synchronization

## Abstract

Cooperation and competition, as two common and opposite examples of interpersonal dynamics, are thought to be reflected by different cognitive, neural, and behavioral patterns. According to the conventional approach, they have been explored by measuring subjects' reactions during individual performance or turn-based interactions in artificial settings, that don't allow on-line, ecological enactment of real-life social exchange. Considering the importance of these factors, and accounting for the complexity of such phenomena, the hyperscanning approach emerged as a multi-subject paradigm since it allows the simultaneous recording of the brain activity from multiple participants interacting. In this view, the present paper aimed at reviewing the most significant work about cooperation and competition by EEG hyperscanning technique, which proved to be a promising tool in capturing the sudden course of social interactions. In detail, the review will consider and group different experimental tasks that have been developed so far: (1) paradigms that used rhythm, music and motor synchronization; (2) card tasks taken from the Game Theory; (3) computerized tasks; and (4) possible real-life applications. Finally, although highlighting the potential contribution of such approach, some important limitations about these paradigms will be elucidated, with a specific focus on the emotional domain.

## Hyperscanning as a tool to assess social dynamics

Cooperation and competition are two common and opposite models of interpersonal exchange (Decety et al., 2004). In fact, according to the interaction type, individuals could facilitate, but also obstruct, others' goal achievement. Nonetheless, the two modalities share some important features. First, from an evolutionary point of view, they are both recognized as human behavioral patterns devoted to survival, although in different ways. Second, they both require some cognitive capacities such as monitoring and mentalizing abilities, to attribute independent mental states, such as thoughts, beliefs, and desires, to others (Flavell, 1999). This allows anticipating and predicting others' intentions and adjusting one's own action accordingly (Decety and Sommerville, 2003). For these reasons, many previous studies focused on these two models as a good example of social and emotional sharing. For example, Decety et al. (2004) asked subjects to participate in couples to a computer game in a functional Magnetic Resonance Imaging (fMRI) scan and compared their neural responses during cooperation and competition. Results highlighted the presence of common networks related to executive functions, as well as a more specific recruitment of different brain areas according to the different mental framework engaged. Also, Liu et al. (2015), by using functional near infra-red spectroscopy (fNIRS), found a differential activation of the right inferior frontal gyrus during cooperation and competition in a turn-taking game. Moreover, Cui et al. (2015) explored the role of these two context in modulating empathy for pain by using event-related potentials (ERP). Finally, Balconi and Pagani (2014, 2015) experimentally manipulated the perceived efficacy during a competitive task to investigate social hierarchies and ranking. However, it has been suggested that the study of social cognition could be reductive and partial by using single-subject or turn-taking paradigm (Schilbach, 2010).

Recent scientific evidence studied these forms of synchronous interactions by considering brain-to-brain coupling. In fact, it has been shown that observing the actions, emotions or feelings of other people can trigger corresponding cortical representations (Hasson et al., 2012), a mechanism defined as vicarious activation (Keysers and Gazzola, 2009). It appears clear that similar processes cannot be captured by conventional experimental approach on individual brains. In the attempt to move a step forward, the hyperscanning paradigm emerged in contrast to previous research approach to allow the simultaneous recording of the neural activation from two, but also multiple, participants interacting jointly (Montague, 2002). This technique permitted to discover typical patterns of inter-brain synchronization during social and emotional exchange thus providing data that can't be obtained by recording single brain activities alone (Babiloni and Astolfi, 2012).

Previous work conducted with imaging techniques such as fMRI allowed identifying the brain areas that are involved during emotional sharing. Nonetheless, fMRI can provide only partial support to this ambitious aim in that it lacks temporal resolution. Also, it is unable to provide a real-time ecological environment in that participants have to lie motionless in a noisy and often emotionally daunting scanner while the verbal communication is discouraged (Cui et al., 2012). Conversely, EEG hyperscanning studies provide higher temporal resolution that permits capturing real-time events. Prior findings showed inter-brain phase coherence across different frequencies, including delta, theta, alpha, beta, and gamma, that can be attributed to a series of different processes, from perception, to cognition, and especially emotion (Balconi et al., 2015). Among the most used techniques are correlation or coherence-based analyses (King-Casas et al., 2005; Funane et al., 2011; Cui et al., 2012), which move from the assumption that the modifications in the activity of certain cerebral regions in subjects can share the same generator/generative source.

Thus, the aim of the present review is to collect and describe existing research on cooperative/competitive dynamics conducted with a hyperscanning approach as a promising paradigm for social neuroscience. Previous reviews already explored the potentiality of such paradigm to social interactions (Dumas et al., 2011; Liu and Pelowski, 2014; Koike et al., 2015), but none of them explicitly focused on these two opposite scenarios, which could provide some precious findings for every-day social life, from work environment, to prosocial behaviors, from collective performance, to affiliation and dyadic bonds. EEG will be valued as a promising technique to capture the sudden and unpredictable modification related to social interactions.

In the next section, the most important evidence in the field will be reviewed and grouped according to the different materials and experimental tasks.

## EEG hyperscanning technique: the case of cooperation and competition

The selection criteria included: use of EEG technique; use of hyperscanning paradigm with real-time interactions; explicit use of cooperative and/or competitive paradigms. According to the different materials and experimental paradigms used to reproduce the social dynamics, available evidence has been grouped in four different categories: paradigms that used rhythm, music, and motor synchronization (section Rhythm, Music, and Motor Synchronization); paradigms based on card tasks taken from the Game Theory (section Evidence from the Game Theory); paradigms based on computerized tasks (section Computer-Based Paradigms); and possible real-life applications (section Real-Life Applications).

### Rhythm, music, and motor synchronization

Some previous studies used rhythmic synchronization to assess the capacity to cooperate each other. Lindenberger et al. (2009) found that, when playing a short melody together, dyads of guitarists showed increased phase synchronized theta and delta oscillations. The authors suggested that coordinated behaviors are characterized by inter-brain oscillatory coherence. Also, since the reported rhythms were all in lower frequency range, it is possible that the similarities in sensorimotor feedback could have enhanced between-brain synchronization.

To disambiguate this issue the same team (Sänger et al., 2012) later used a similar but advanced paradigm with a more complex piece of music such that the two members of the couple would have different roles, a leader, and a follower. The paradigm reduced similarities in movement, proprioception, and perception. Results extended previous data and attributed between-brain phase coherence to musical coordination periods. Also, since the effects were larger at frontal and central sites, it was proposed that the on-line representation of one's own and others' actions and their combination into a joint, coupled model, may help supporting interpersonal action coordination (IAC).

A recent finger-tapping experiment replicated this asymmetrical pattern in leader-follower dynamics (Konvalinka et al., 2014): it was demonstrated that it is possible to differentiate roles on the basis of the modulation of frontal alpha-suppression, being this latter prominent in leaders than followers. It has been hypothesized that leaders probably allocated more resources to self-processing to monitor their own rhythm, while followers should monitor the output of their partner.

Analogously, another study by Yun et al. (2012) used a leader-follower task to demonstrate the presence of implicit motor synchronization when interacting with another human. Seated face to face, a leader had to perform hand movements and another player had to imitate them at their best. Finally, both participants were asked to freeze. The behavioral results highlighted that the two mates implicitly synchronized their movements, mainly during the final phase that followed imitation. EEG results showed higher phase synchronization following the imitation phase within theta and beta frequency bands over the inferior frontal gyrus, anterior cingulate, parahippocampal gyrus, and post-central gyrus. Such results were considered as an improved coupling between the two cognitive representations.

Similarly, Dumas et al. (2010) used a video feedback system and asked subjects to imitate the other's hands movement. The researchers found higher inter-brain phase synchronization within mu, beta, and gamma range in the right centro-parietal areas of the two brains during behavioral synchrony.

**Figure 1:**
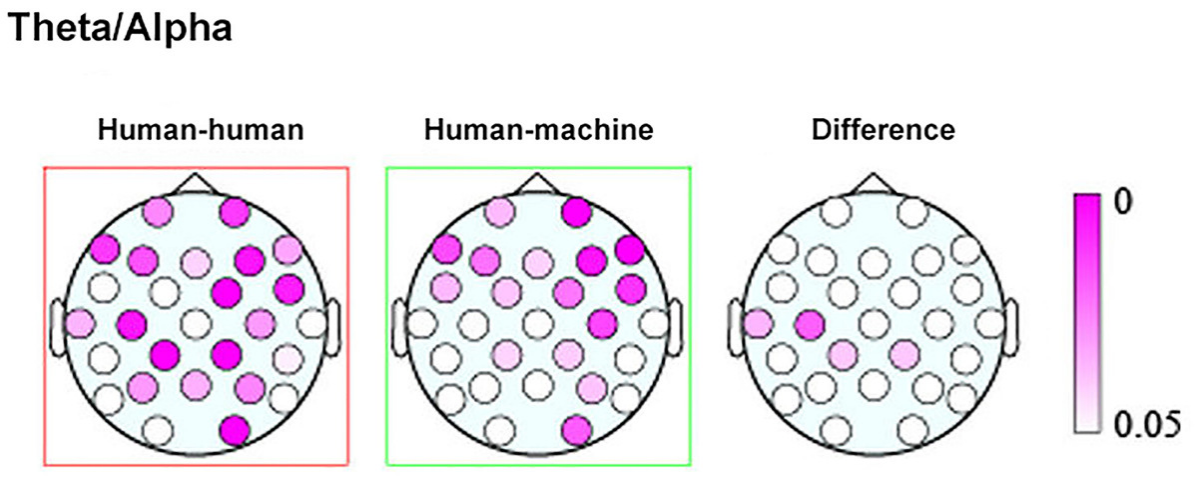
Topographic maps showing the *p* values of the theta/alpha amplitudes during human–human **(left)** and human–machine **(center)** conditions, as well as their difference **(right)**. Taken and modified from Kawasaki et al. (2013).

Finally, a work by Kawasaki et al. (2013) explored the presence of inter-brain correlation during speech rhythm synchronization. Results showed that speech rhythms were more easily synchronized in the joint condition with respect to the individual condition where subjects performed the same task within a computerized session. Moreover, increased synchronized theta/alpha amplitudes were found in the same temporal and lateral-parietal regions known to be associated with social cognition, such as comprehending others' intentions, affects, and actions (Adolphs, 1999) (Figure 1).

The mentioned studies are relevant to neuropsychophysiology since they show how neural synchronization can emerge and be studied with simple matched behaviors involving motor and rhythmic coordination. Moreover, it has been shown that EEG technique can recognize the different roles assumed within the couple. In fact, the cognitive and behavioral states related to the joint task can modulate rhythm synchronization.

### Evidence from the game theory

A series of studies conducted by Astolfi et al. (2009, 2010, 2011b,c) used the Prisoner's Dilemma paradigm: a cooperation/competition task that requires to decide whether to cooperate or defect. The game requires two players (or groups) and two alternative choices: cooperate or defect. When both players decide to cooperate, they both gain small wins (cooperation condition). If only one player cooperates and the other retracts, the cooperator obtains a big loss and the defector a big win. If both players betray, they have small losses (defeat condition). The aim of the game is to gain the highest score. Through this sharp paradigm the research group obtained some important results: first, the defeat conditions elicited the higher cortical activity in the theta and alpha frequency band. This choice, in fact, can be related to major penalty and risky conditions when compared to cooperation. Also, this effect was mostly present over the frontal regions, in accordance with the decisional request (Astolfi et al., 2009, 2010).

A successive study with the same paradigm (Astolfi et al., 2011c) integrated such data with functional connectivity analyses and found that the pattern of inter-brain connectivity in the cooperation condition is denser than in the defect one. In fact, as an individualist act, the defect choice could produce a lower synchronization between brains. On the other hand, a cooperative act could elicit weaker brain activity, but a denser synchronization between the two brains.

Research coming from the Game Theory tradition is relevant in that provides a standardized tool to directly compare cooperation and competition, but also different studies each other. Thus, it was possible to differentiate the two conditions, associating cooperation with increased neural connectivity between the two brains resonating each other.

### Computer-based paradigms

A series of hyperscanning studies used computer-based paradigm to assess cooperation and competition in experimental settings. For example, Astolfi et al. (2014) asked participants to lift a rolling ball up to a particular target region placed at the top of the screen with a virtual bar. There was a joint condition, where both subjects played together on the same task, a solo condition, where both subjects were asked to complete the task individually, and a PC condition which was identical to the joint one, but subjects were told that they were playing against a computer. The comparison between joint and PC, as well as between joint and solo condition, revealed significant differences in terms of inter-brain functional causal relations.

In another study by Sinha et al. (2016) the authors investigated the effect of cooperative and competitive interactions with a game similar to table tennis. The aim is to defeat the competitor by striking a ball back and forth using a vertical bar (competition condition) or to act as a team to defeat a computer program (cooperative condition). Results showed that the cooperative condition was characterized by significantly higher synchronization as compared to competition.

Another computer-based task was proposed by Balconi and Vanutelli (2016) within a competitive scenario where participants coupled in dyads had to perform better than their opponent in a sustained-attention task. During the game they were continuously informed about their performance and, halfway through the task, they received a general feedback reinforcing the results obtained so far and the instruction for the second part of the game. The analyses showed a systematic response within the prefrontal regions (PFC) during competition. This effect was mainly present after receiving a positive feedback assessing a good performance and a winning situation. Also, considering the enhanced PFC responsiveness, a specific lateralized pattern was found in favor of the left hemisphere, compatible with positive emotions, and approach-related motivations. Accordingly, winners' behavioral performance was improved in terms of reduced reaction times (RTs).

With respect to card games, computer-based hyperscanning studies offer more controlled, even if less ecological, paradigms to study cooperation and competition. Also, they allow varying the experimental conditions according to specific research aims. In particular, it is possible to manipulate the cognitive scenarios to induce different and correspondent neural synchronization as in the last example (Balconi and Vanutelli, 2016) where the affective state could modulate both neural activation and performance.

### Real-life applications

**Figure 2:**
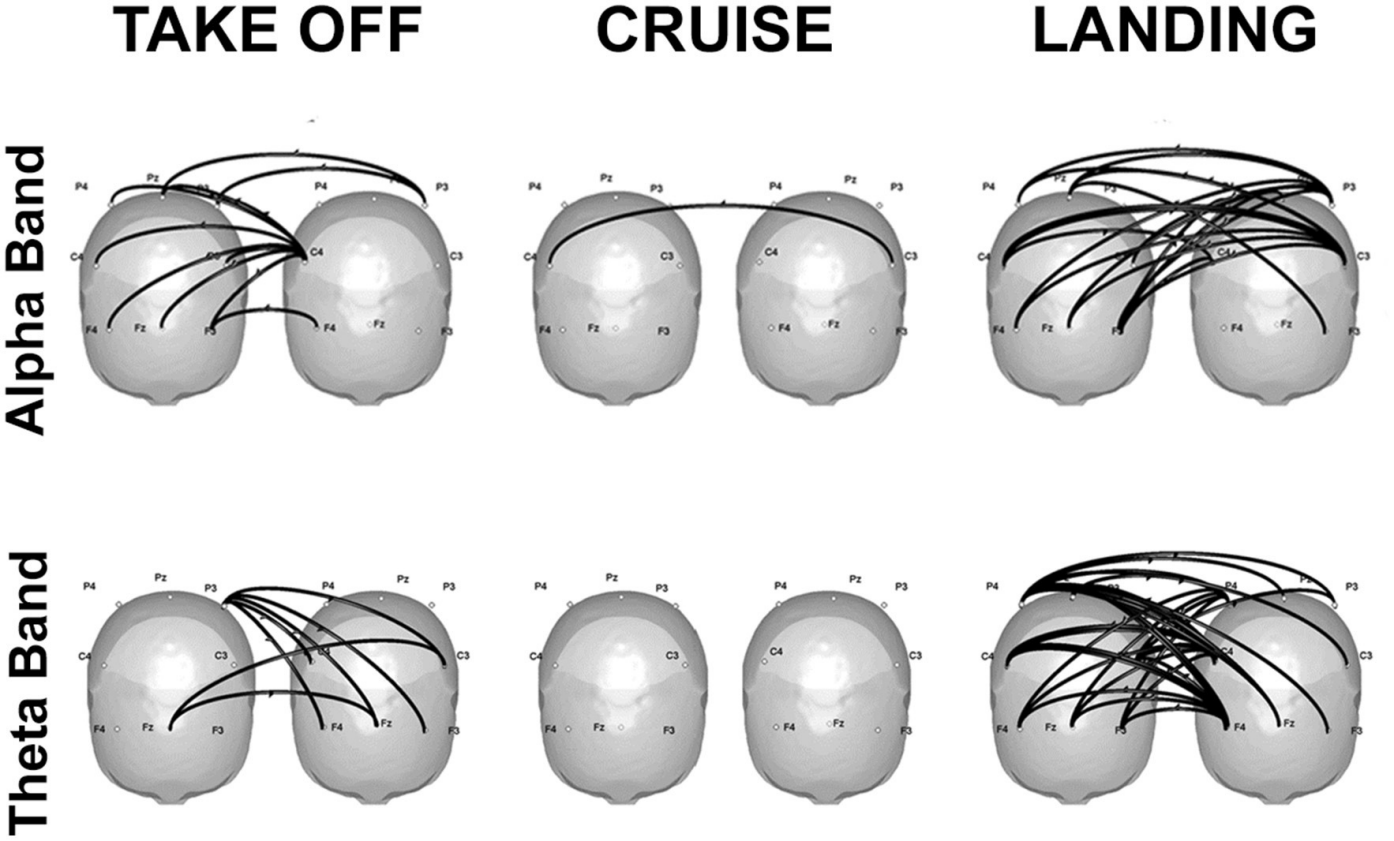
Significant connectivity elicited in the alpha **(top)** and theta **(bottom)** frequencies during takeoff **(left)**, cruise **(center)**, and landing **(right)** of one exemplificative couple composed by the first officer **(left)** and the captain **(right)**. Taken and modified from Toppi et al. (2016).

Finally, some promising real-life applications through hyperscanning methods are reviewed: a first contribution refers to flight simulations in couples of pilots and co-pilots (Astolfi et al., 2011a, 2012; Toppi et al., 2016). Results showed increased coherence in the alpha band over the parietal sites during the most demanding phases of the simulation, which can be attributed to higher cognitive load, as well as in the theta band over the frontal sites, which is compatible with increased resources engaged for information processing (Klimesch, 1999). Hyperconnectivity patterns linking frontal and parietal areas of the two participants emerged during the phases involving a close interaction between the two pilots, that is takeoff and landing. In particular, the strongest connections were located over the frontal sites, and were directed from the co-pilot toward the pilot (Figure 2).

Finally, an innovative application was proposed by Balconi et al. (Venturella et al., 2017) within a neuromanagement approach: the authors proposed a pilot study on the brain dynamics occurring during a role-played employees' evaluation in couples of manager-collaborator. Preliminary results showed greater delta and theta response to positive and constructive inter-subjective exchange, as well as to the conversational moments while sharing the company mission and aims.

Such examples are particularly relevant in that they can be used to get neuroscience closer to real-life situations and to improve specific work environment where the performance depends on good cooperative/competitive dynamics. In fact, it has been demonstrated that specific phases or topics during dyadic work simulation can be identified by specific neural markers which can be indicative of higher or lower cognitive demand, emotional involvement and interactive skills.

### Methodological and statistical caveats

However, how were these results obtained? Being a very complex and innovative paradigm, a few methodological and statistical considerations about hyperscanning should be discussed. First, hyperscanning conventionally means both the experimental paradigm including the simultaneous registration of multiple brain activities, and/or the specific connectivity analyses performed on resulting multiple data. In this second case, the most used techniques are based on correlation or coherence analyses (King-Casas et al., 2005; Funane et al., 2011; Cui et al., 2012). Since the computation is made on time series, the paradigm should include a high number of frames for each experimental condition. This issue can be solved by (a) using EEG or other techniques which can provide a high sampling rate: the higher, the better; (b) reducing the experimental factors. For example, the number of brain regions could be simplified by creating regions of interests (ROI), or performing specific computations such as principal component analysis (PCA). Finally, since couples are the cases for statistics instead of single subjects, the number of participants should be improved. Anyway, to solve these criticalities, before performing the experiment a power analysis would be recommended.

## Emotions in hypescanning studies: the big absentee

As already discussed in previous sections, EEG-based hyperscanning technique provides a valid and innovative tool for exploring coupled responses and obtaining real-time results in highly-ecological paradigms. Nonetheless, it seems that most experimental paradigms did not explicitly taken into account the affective component (Acquadro et al., 2016) in terms of emotional contagion, sharing, and social exchange. Moreover, the pioneeristic nature of these studies often led to adopt an explorative approach and, accordingly, to vague and sparse findings.

However, previous research on both animals and humans has suggested that the psychophysiological connection between two individuals is an intrinsic element of affective bonding (Coan et al., 2006; McAssey et al., 2013). In fact, when we interact with someone else, our brains and bodies can no longer be considered independent, but must be viewed as part of a new environment with the other person, in which we become coupled through a continuous and mutual adaptation (Konvalinka and Roepstorff, 2012). Besides neural synchronization, such dynamic and interactive process has been also shown to result in an alignment of behavior (Konvalinka et al., 2010), posture (Shockley et al., 2003), autonomic systems such as respiration (McFarland, 2000; Giuliano et al., 2015) and cardiac rhythms (Konvalinka et al., 2011; Müller and Lindenberger, 2011).

For these reasons, it should be important that hyperscanning paradigms would also consider the affective components related to cooperative and competitive scenarios, and, possibly, to combine other autonomic or behavioral measures (Niedenthal, 2007; Keysers et al., 2010).

From a clinical point of view such results are particularly relevant. In fact, such inter-personal couplings generate social bonds that could facilitate or obstruct future successful exchange. For example, higher synchronization in heart rate variability is associated with the length of romantic relationship (Anderson et al., 2003). On the contrary, few developmental studies found that mother–child synchrony decreases in particular conditions (Feldman, 2007).

Thus, the adoption of clear theoretical approach and specific research questions about the role of emotions in modifying neural and bodily synchronization would help designing hyperscanning protocols with different emotional conditions or clinical groups to be compared. Accordingly, the methods could be refined by including some subjective factors such as the motivation in participating to the task, the effective involvement in the role or the experimental condition, but also all those psychological variables which could differentiate subjects or couples by their personality, affective style, dominance, and so on. To conclude, the need for experimental situations leading to emotional engagement is still urgent in a way to enhance the understanding of emotions within social interactions, and improve the ecological validity of cooperative and competitive settings.

## Author contributions

MB and MV critically discussed the literature and wrote the paper.

### Conflict of interest statement

The authors declare that the research was conducted in the absence of any commercial or financial relationships that could be construed as a potential conflict of interest.
